# Dissecting meiotic recombination based on tetrad analysis by single-microspore sequencing in maize

**DOI:** 10.1038/ncomms7648

**Published:** 2015-03-24

**Authors:** Xiang Li, Lin Li, Jianbing Yan

**Affiliations:** 1 National Key Laboratory of Crop Genetic Improvement, Huazhong Agricultural University, Wuhan 430070, China; 2 Department of Agronomy and Plant Genetics, University of Minnesota, Saint Paul, Minnesota 55108, USA

## Abstract

Meiotic recombination drives eukaryotic sexual reproduction and the generation of genome diversity. Tetrad analysis, which examines the four chromatids resulting from a single meiosis, is an ideal method to study the mechanisms of homologous recombination. Here we develop a method to isolate the four microspores from a single tetrad in maize for the purpose of whole-genome sequencing. A high-resolution recombination map reveals that crossovers are unevenly distributed across the genome and are more likely to occur in the genic than intergenic regions, especially common in the 5′- and 3′-end regions of annotated genes. The direct detection of genomic exchanges suggests that conversions likely occur in most crossover tracts. Negative crossover interference and weak chromatid interference are observed at the population level. Overall, our findings further our understanding of meiotic recombination with implications for both basic and applied research.

## Abstract (web summary)

The crossovers and gene conversions that occur during meiotic recombination contribute to genome diversity in eukaryotes. Here Li *et al*. describe a method of isolating individual microspores for whole-genome sequencing, providing new insights into the generation of genome diversity through sexual reproduction.

## Graphical abstract

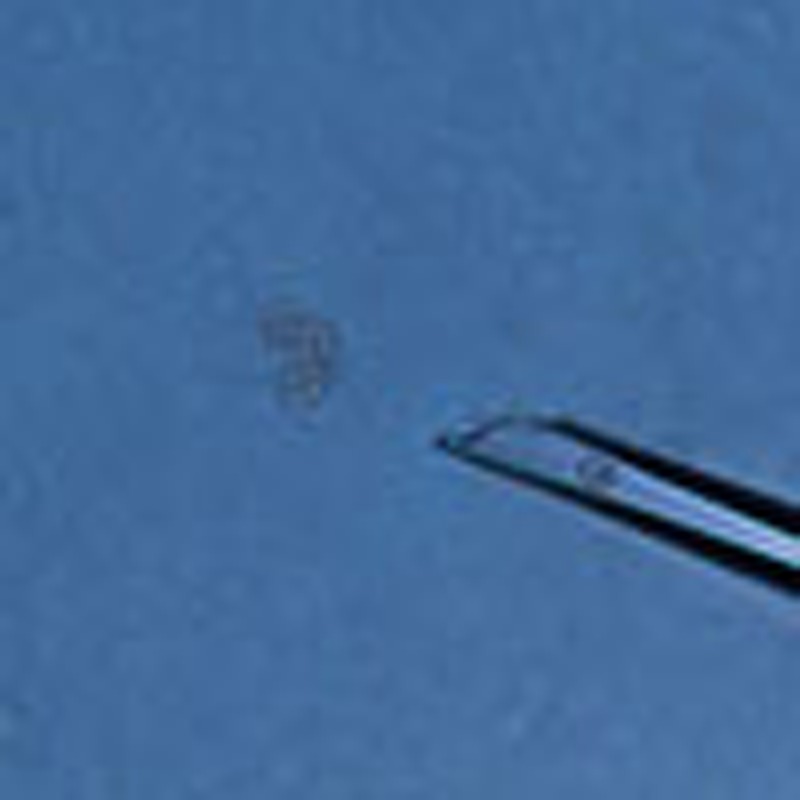

Meiosis produces haploid gametes from parental diploid cells in sexual reproduction. During prophase I of meiosis, chromosome double-strand breaks are initiated and repaired by homologous recombination between chromatids and result either in genomic exchanges (crossover, CO) or non-exchanges with synthesis-dependent strand annealing (non-crossover, NCO). Both CO and NCO may give rise to gene conversions (GCs), the non-reciprocal genomic exchange between homologous non-sister chromatids, which results in the generation of new alleles. COs reshuffle parental alleles and generate new allelic combinations in the gametes via double-Holliday junction exchange and repair between non-sister chromatids (DNA double-strand break repair), whereas NCOs accompanied by GC can generate new alleles in an otherwise unchanged background by synthesis-dependent strand annealing^1^. Consequently, there would be both 2:2 (with CO) and 3:1 (with CO and NCO accompanied by GC) segregation of alleles among the four gamete cells of a single meiosis. Meiotic recombination could also ensure proper chromosome segregation by stabilizing the bivalents via formation of chiasmata^2^,^3^. Meiotic recombination thus plays an important role in the genetic diversity by contributing to allele assortment, creating a substrate for natural selection^4^ and evolution of eukaryotic genomes^5^.

The rate and distribution of meiotic recombination events largely determine allele distribution and haplotype structure in the offspring population. The rate of recombination shows marked intraspecific^6^,^7^ and interspecific^8^ variation, which is related to genome composition in almost all organisms studied to date^4^,^9^. Historically, understanding of recombination was aided by observing segregating populations with more than one generation of meiosis. In yeast, recombination is well understood because the four haploid progeny resulting from a single meiosis are held together within an ascus. These can be separated and the individual meiotic progeny can be clonally propagated such that their genomes can be sequenced without amplification^10^,^11^. In Arabidopsis, the *qrt1* mutant maintains mature pollen grains from a tetrad together, and is regarded as an ideal genetic system to directly analyse the products of a single meiosis^12^,^13^,^14^,^15^. With the advent of next-generation sequencing technology and the whole-genome amplification of a single cell, it is feasible to study recombination at the gamete level and visualize single-meiotic events at nucleotide-level resolution^16^. In humans, studies of sperm, eggs and polar bodies by single-cell next-generation sequencing provided a robust strategy to directly dissect meiotic recombination at ultrahigh resolution^17^,^18^,^19^. In plants, single-cell sequencing is still challenging because the cell wall hinders the isolation and lysis of the nuclear contents. As a cytogenetic and genetic model, maize has been successfully used for the dissection of recombination variation^6^,^7^,^20^,^21^,^22^.

Here we describe a simple method to isolate and sequence the whole genome of each of the four microspores from a tetrad to facilitate the study of recombination at the single-cell level in plants. A high-resolution recombination map was constructed from 24 tetrads by using 599,154 single-nucleotide polymorphisms (SNPs). The results reveal that COs were unevenly distributed across the genome, and more likely to occur in the genic than intergenic regions. GCs were directly detected and seem exist in most CO tracts. Negative CO interference was observed that means double CO frequency is significantly greater than expected. Complex chromatid interference was also first observed in maize, which implies that the genetic background may affect genomic selection and evolution. These findings provide beneficial information for better understanding of meiotic recombination thus enhancing plant breeding.

## Results

### Single-microspore sequencing of a maize tetrad population

**Figure 1:**
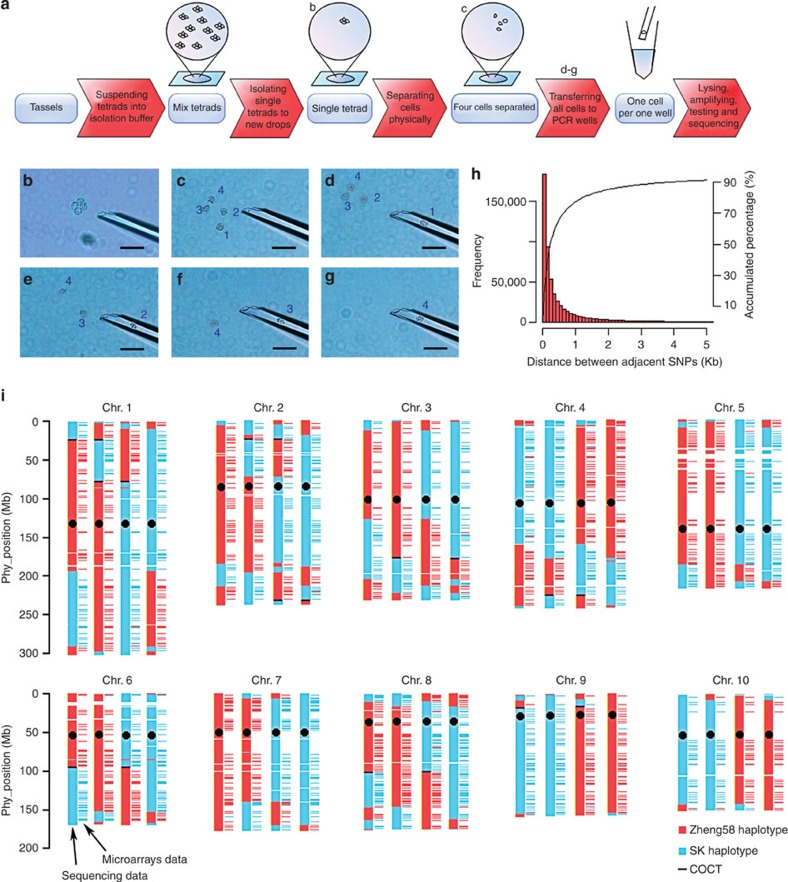
Single-microspore sequencing in plants. (**a**) Schematic diagram of single-microspore isolation. (**b**) Tetrads from tassels are suspended in buffer, and single tetrads are isolated into a new drop of buffer with a tiny glass pipette. (**c**) Four single microspores are immediately separated by aspirating and blowing them out. (**d**–**g**) Extracted cells are individually placed into PCR wells for lysis and MDA amplification (scale bar, 100 μm; 1–4 mean the four microspores) (**h**) A high-resolution SNP data set is obtained and high-quality SNPs are identified by filtering with multiple criteria. The histogram and the cumulative per cent distance between adjacent SNPs show a high coverage of sequencing data. (**i**) The haplotypes of tetrad 17 based on sequencing and SNP chip, share a consistent recombination pattern, indicative of the high quality of data generated by this method. COCT, CO-associated conversion tract.

F_1_ maize hybrid individuals from a cross between Zheng58, an elite inbred that has been deeply sequenced previously^23^, and SK, a tropical inbred selected from a landrace population, were grown. Products of cell fission during pollen development contain meiotic process from microsporocyte to tetrad stage, mitotic process from microspore separated to mature pollen stage. In this study, we were able to capture the intact tetrad even tally separate into individual microspores before separation by carefully timing the isolation process during normal development the tetrads. The four microspores from the same developing tetrad were isolated manually under the microscope using a glass micropipette system (Fig. 1a–g). Because of high internal osmotic pressure, the cells were submerged in 27% D-sorbitol solution during the isolation process. Repeated aspirations were used to destroy the cell wall and disrupt the tetrads. The four individual cells were then placed in PCR tubes and subjected to lysis. The multiple displacement amplification (MDA) method^24^ was used to amplify whole-genomic DNA. The coverage of MDA products was estimated using 10 molecular markers (Supplementary Fig. 1); 96 microspore cells from 24 tetrads were selected for further analysis. Preliminary genotyping with 3,072 SNPs was conducted to validate the integrality of 12 randomly selected tetrads. Successful genotyping of 42–62% of these SNPs with very few (<1%) heterozygotes indicated that high-quality DNA was obtained by the MDA method, sufficient for whole-genome sequencing (Supplementary Table 1).

The 96 microspores were sequenced at ~1.4 × genome depth by the Illumina Hiseq 2,000 platform to obtain a total of 3.8 billion reads covering ~41% of the maize genome on average. Approximately 92% of the filtered sequencing reads were aligned to the maize B73 genome (Supplementary Data 1). We identified 1,269,588 raw SNPs between the parents. A final count of 599,154 high-quality SNPs were obtained following filtering by a strict procedure with multiple criteria and used for further analysis (see method, Supplementary Table 2). An average of 271,524 SNPs were available per tetrad, with a range of 124,957–335,266 SNPs (Supplementary Data 1). These SNP sets had a median distance of 235 bp between consecutive markers on each chromosome (Fig. 1h,i), providing the opportunity to characterize recombination patterns at a very high resolution.

### High-resolution recombination landscape

In contrast to other types of segregating populations such as recombinant inbred lines (RILs), we could directly estimate COs during a meiotic process by identifying genomic exchanges among the four chromatids of the same tetrad (Fig. 1i). The overall recombination pattern was found to be largely in agreement with that obtained by low-density SNP array studies (Fig. 1i), supporting high data quality. A total of 924 COs were detected in the 24 tetrads (Supplementary Data 2), ranging from 24 to 50 in different tetrads (Supplementary Table 3) with an average of 38. This is considerably higher than the 20.5 COs per maize microsporocyte reported previously^20^. This may be due to higher genetic diversity^6^ and/or CO interference (see below). The average CO numbers of tetrads collected from the two F_1_ individuals were also significantly different (*P*=4 × 10^−4^, analysis of variance), with 31.6±5.6 from individual 1 (*n*=7) and 41.4±5.0 from individual 2 (*n*=17) (Supplementary Table 4). The number of COs in haploid daughter cells ranged from 8 to 29 (Supplementary Data 3).

**Figure 2:**
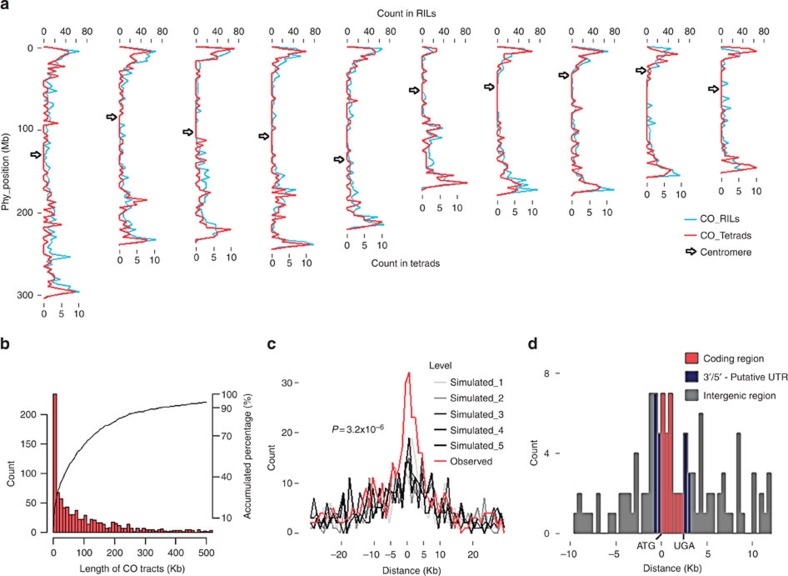
Non-uniform crossover distribution in maize. (**a**) The crossover distribution pattern by using a window size of 3 Mb based on the B73 reference genome. (**b**) The histogram and cumulative per cent of CO tract length. (**c**) The frequency plot of distances from CO to ATG of the closest transcripts. The observed COs (in red) are significantly concentrated around genes, compared with the absence of COs in intragenic regions in the five simulated sets (in black and grey). (**d**) The crossover distribution at the gene level. In the frequency plot (**c**), there is a significant difference (Kolmogorov–Smirnov test, *P*=3.2 × 10^−6^) between observed set and five all simulated sets. In the frequency plot (**c**) and the histogram (**d**), the ‘Distance’ from COs to ATG of the most closed transcripts is not the physical distance, but normalized scale, proportionally changed as we scaled the annotated lengths between ATG and UGA to the average, 2.5 Kb. For example, physical distance was divided by the length between ATG and UGA of the most closed transcripts, then multiplied by 2.5 kb. In the histogram (**d**), we also assume that the length between ATG and UGA accounts for 60% (six columns in red) of transcript. The 3′/5′-untranslated region (UTR) represents the other 40% (two columns each in midnight-blue) of it.

To detect the distribution of COs across the whole genome, we first determined CO distribution along each chromosome and found that the CO number in each chromosome is positively correlated with the chromosome length and the length of the synaptonemal complex (Supplementary Fig. 2; Pearson *r*=0.95 and 0.98, respectively), which is in accordance with previous reports^25^,^26^. The CO number was then evaluated at the genome level. As expected, COs were more likely to be located in the arms of each maize chromosome and the number of COs decreased towards pericentromeric regions (Fig. 2a). However, it is worth noting that there are a few pericentromeric regions having high frequency of COs, which is contradicting the expectation. Such non-random distribution of COs is highly concordant (Pearson *r*=0.72; *P*=2.0e−16, Fig. 2a) with that of the RIL population derived from the same parents (SK and Zheng58) and genotyped with the maize SNP50 beadchip^27^.

High-coverage SNPs could accurately define COs, of which 80.2% (741) were located in an interval of <200 Kb (Fig. 2b). The size of some CO interval were still large due to the following: (1) the region of the genome may not have sufficient genetic diversity (SNPs that differ between the two parents) or (2) the sequence coverage in the region of specific sample may be poor. To avoid possible errors, we only focused on the 581 CO tracts localized to <100 Kb to define the location of COs relative to genes. A high number of COs was observed close to the ATG initiation codon positions of genes. No similar high numbers were seen in the same regions in the five simulated repeats (Kolmogorov–Smirnov test, *P*=3.2 × 10^−6^; for details see Methods), indicating that COs are more likely around genes (Fig. 2c). This result is similar to CO studies in yeast^28^ and Arabidopsis^29^, but differs markedly from CO studies in humans^18^,^19^,^30^, where COs decreased towards gene transcripts. We then focused on COs within tracks of 10 Kb or less (234, 25.3%) to determine the exact location of COs relative to gene models (from B73 reference genome AGPv3.21, annotated by maize genome sequence project^31^). COs were consistently more likely to occur at the 5′ end of genes, followed by the 3′ end (Fig. 2d). This suggests that genomic exchanges driven by recombination in maize tend to alter the promoter/regulatory regions (5′ and 3′ ends) of genes and may orchestrate gene expression changes in progeny.

### GC occurs in most COs

During GC, DNA sequence from a donor chromosome is transferred to a homologous acceptor region between sister chromosomes or chromatids. GC is evolutionarily important, especially for DNA repair during recombination^32^,^33^,^34^. The CO-associated conversion tracts (COCTs) were reported to be <1-kb long in Arabodopsis^35^ and 2 kb in yeast^10^. To date, GC has been scarcely studied at the genome level in maize due to the limitation of small GC length and the lack of high-resolution techniques and suitable genetic materials (for example, *qrt1* mutant in Arabidopsis^12^). In the present study, we examined 924 COs, of which 160 contain tracts segregating in non-Mendelian ratios (3:1) and assumed to be subject to GC events (Supplementary Data 2). This number of GC events may be an underestimate, due to the limited sequencing coverage, short tract length and polymorphism between the two parents. Of the 160 COCTs, 10 were fine-mapped to <10 Kb genomic regions. Seven COCTs had the SK donor segments, while only three had Zheng58 donor segments, suggestive of potential parental conversion bias but due to small sample size, is inconclusive (*χ*^2^-test, *P*=0.07).

**Figure 3:**
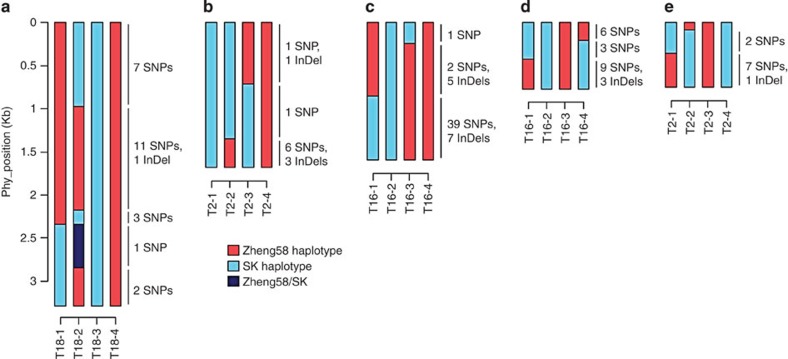
Validation of COCTs by Sanger sequencing. Five crossover tracts were randomly selected from those fine-mapped in a narrow genomic region (<4 Kb). Gene conversions have been validated in all five tracts by Sanger sequencing. The start positions (0 bp) of these regions resequenced (**a**–**e**) are chr4:129,510,201; chr6:160,235,403; chr4:9,636,448; chr6:115,545,843; chr1:4,350,391, respectively.

Five CO tracts (including two COs without detectable conversion estimated by high-throughput sequencing) were selected for validation by Sanger resequencing. One GC regions only have one SNP detected, which may be false discovery. Intriguingly, GCs were identified in all five tracts and the GC tract length ranged from 220 to 1,875 bp (Fig. 3a–e). These results follow the hypothesis that GCs are coupled with CO events based on the observation that double-strand break repair and mismatch repair could result in a non-Mendelian segregation ratio for at least one tract segment^1^. Consequently, GCs should be frequently detected during COs. Moreover, the complex COCT with the largest GC track length (1,875 bp) was observed with normal DNA segments and one unexpected heteroduplex DNA segment (Fig. 3a). This indicates that the heterozygous double-strand break region may not have been repaired completely until the tetrad stage. However, since only one heterozygous SNP was identified in this region, this could be an error^36^ during the whole-genome amplification process, which occurs with a rate of 10^−6^–10^−7^.

### Large-scale negative CO interference in maize

**Figure 4:**
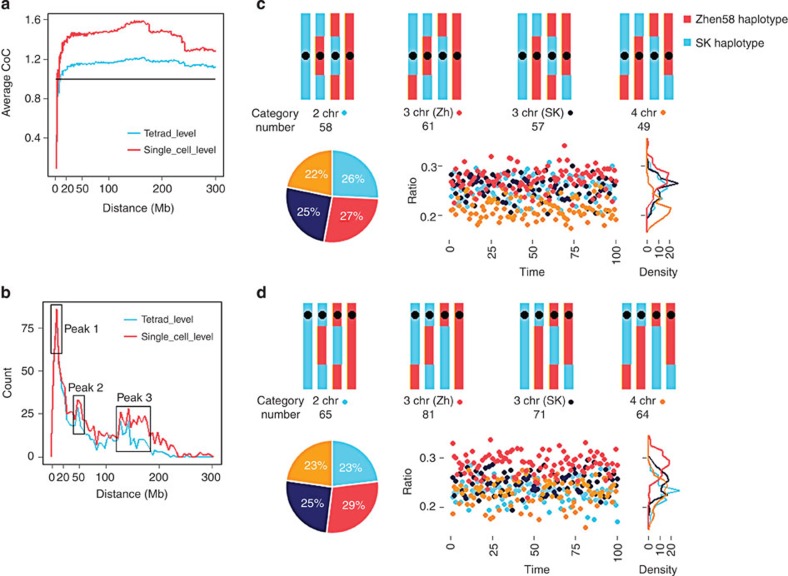
Genetic interference. (**a**) Crossover interference. The average CoC calculated in 1-Mb windows show an expected positive interference (<10 Mb), as well as an unexpected negative interference (beyond 10 Mb) at the chromosome level. The horizontal line represents the expected level without inference (CoC=1). (**b**) Distance distribution between adjacent crossovers. Three peaks were observed at 10, 50 and 120–180 Mb intervals of adjacent crossovers. (**c**,**d**) Chromatid interference. We define four categories, and evaluated them at two levels—two COs happen between the two arms of a chromosome (level 1, **c**) and along the same arm of a chromosome (level 2, **d**). The ratio and bootstrapping analysis suggested that chromatid interference exists in both the levels (significantly different from the results in human studies). Bootstrapping procedures randomly selected 15 of 24 tetrads 100 times, and the ratios of the four categories were calculated for each.

Crossover interference manifests itself in the decreased observation of two CO events occurring in close proximity on a chromosome. The coefficient of coincidence (CoC) is a measure of the strength of CO interference^37^,^38^. We calculated the CoC within a 1-Mb window across the whole genome (Fig. 4a) and identified strong negative interference between pairs of random sites at the whole-chromosome level, except for pairs of sites <10 Mb apart. This suggests a relatively narrow window of 10 Mb as the point of transition between positive and negative interference (Fig. 4a). Compared with previous results in humans^19^, yeast^39^, mice^40^ and *Arabidopsis*^41^, maize has positive interference at shorter distances, and negative interference increasing the number of COs at >10 Mb apart. The average CoC is 1.15 at the tetrad level (1.43 in a single cell), indicating that the observed CO rate is 0.15-fold higher than expected in each tetrad and 0.43-fold higher in a single cell. Because CO interference rate has been found to differ among maize populations^6^, negative CO interference may contribute to the larger number of COs seen in this study than previously reported^20^ (see above). Our data also allowed us to map CO intervals precisely. The distribution of intervals between adjacent COs on the same chromosome had three clear maxima, at ~10, ~50 and 120–180 Mb (Fig. 4b). Moreover, at recombination active regions, the probability of COs occurring at <10 Mb from each other was higher (Supplementary Fig. 3a). Non-uniform distribution of recombination active regions (Supplementary Fig. 3b) may be one of reasons underlying negative CO interference (Fig. 4b).

### Weak chromatid interference detected in the maize genome

In the absence of interference, CO distribution on all chromosomes would be random. Previous studies have found that humans^19^ and fungi^42^,^43^,^44^,^45^ have different levels of chromatid interference. Since all four microspores from a tetrad were isolated in the present study, we were able to analyse chromatid interference in plants. To analyse chromatid interference accurately, we defined four categories of chromatid COs as follows: ***2 chr***, COs among two chromatids (one from parent SK and one from parent Zheng58); ***3 chr (Zh)***, COs among three chromatids (two from parent SK and one from parent Zheng58); ***3 chr (SK)***, COs among three chromatids (one from parent SK and two from parent Zheng58); and ***4 chr***, COs among four chromatids (two from parent SK and two from parent Zheng58) (Fig. 4c,d). These categories were investigated at two levels between the two arms of a chromosome (level 1, Fig. 4c) and along the same arm of a chromosome (level 2, Fig. 4d). The observed number of COs was 58, 61, 57 and 49, respectively, for the four categories defined above in level 1. Unexpectedly, bootstrapping analysis indicated that the ratio of the four categories at level 1 is 36.1±3.2:38.0±2.8:35.7±3.1:30.8±3.0, which is significantly different (*P*=8.14 × 10^−56^ for 100 times bootstrapping analysis) from the expected ratio of 1:1:1:1 for random distribution (Fig. 4c). The least abundant category (***4 chr***, 22%) is correlated with the difference of CO interference between the tetrad and single-cell levels. Only when ***4 chr*** is <25% can CoC at the single-cell level be higher than at the tetrad level. The observed number COs was 65, 81, 71, and 64, respectively, for the four categories defined above in level 2. This is also a significant deviation from the expected ratio of 1:1:1:1 for random distribution with an observed ratio of 41.5±4.4:49.4±4.2:44.2±3.6:40.2±4.2 based on 100 times bootstrapping analysis (*P*=1.04 × 10^−59^; Fig. 4d). This result implies that chromatid interference may exist not only within one chromosome arm, but also between arms, unlike observations in humans^19^. Moreover, the proportion of the ***3 chr*** (***Zh)*** COs and ***3 chr (SK)*** COs varied in both the levels suggesting that different genetic backgrounds probably influence chromatid interference and results in parental bias during chromatid COs. Different fungi data sets of the same species also show different degrees of chromatid interference^43^,^44^,^45^, indicative of the effect of genetic background in other kingdoms as well.

## Discussion

Isolation of genetic material for whole-genome analysis at the single-cell level is challenging in plants, which have rigid cell walls. We successfully isolated and sequenced all four microspores from 24 individual tetrads despite the presence of the plant cell wall, by developing a simple physical isolation method. This approach allowed us to construct a near nucleotide-resolution landscape of recombination in tetrad populations of maize. We obtained about 41% maize genome coverage with ~1.4 × sequencing, a little higher than reported for single sperm^18^ (23%) and oocyte^19^ (32%) sequencing in humans, with comparable sequencing depth. The developed isolation method may also apply in other flowering plants.

Tetrad analysis is the ideal genetic technique for accurately analysing meiotic recombination, CO/NCO-associated GCs and genetic interference^11^. However, whole-genome tetrad characterization has only been accomplished in a few species, especially in yeast, in which all meiotic products are kept together as spores in an ascus and are easily isolated^10^ or in the Arabidopsis *qrt1* mutant, which retains intact tetrads^13^. Here we developed a method to analyse the four meiotic products of normal maize tetrads and described a high-resolution map of meiotic recombination. We observed an average of 3.85 COs per chromosome per meiosis, greater than the 1.8–2.0 COs per chromosome per meiosis in *Arabidopsis*^35^,^46^ and less than the 5.66 COs per chromosome per meiosis in yeast^10^. Detailed comparisons among previously studied species are listed in Table 1. It is interesting that average CO numbers varied significantly (31.6 versus 41.4, analysis of variance *P*=3.7 × 10^−4^) in two maize F_1_ individuals. The genotypes of the two individuals are identical, but the tetrad cells collected on different days and under different environmental conditions. This naturally suggests an environmental role in the variation noted for meiotic recombination. Previous study in *Arabidopsis* also documented elevated CO frequencies following growth in higher temperatures^13^. This all suggests that increased recombination frequency may be obtained by varying environmental conditions, and this outcome may be highly desirable in a breeding program to generate more selectable variation. However, since only a small population (7 versus 17 tetrads) was used and also lack additional evidence, further experiments with big population size and in multiple environments are required to exclude that the phenomenon is not due to stochastic variation.

GC involves the unidirectional transfer of DNA sequence from a ‘donor’ chromosome to a highly homologous ‘acceptor’ and was found to be associated with human inherited disease^47^. The role of GC has not been well studied in plants. We found that nearly 20% of COs contained GCs in the 24 maize tetrads examined. This was further validated in five COCTs by Sanger sequencing. In a recent study of *Arabidopsis*, up to 265.3 GC tracts per meiosis were identified^48^, which is much higher than in the present study and also higher than in other *Arabidopsis* studies^14^,^35^,^46^. The rate of GC in humans was estimated to range from 0.3 to 10, also suggesting that CO rate varies among different genetic backgrounds in animals^41^. More recently, study of the *bz* locus showed most recombinants were attributed to GC^49^. The phenomena of polarized distribution of recombination initiation sites within the *bz* locus is very similar to our present finding that recombination at the genomic level occurs with high frequency at the 5′ and 3′ ends of genes (Fig. 2d). We believe the frequency of GC events in the present study is underestimated, since the low sequencing coverage depth limited the ability to identify smaller CO- and NCO-associated GCs. We were unable to identify NCO-associated GCs because the length of each tract containing an NCO-associated GC could be as small as tens of base pairs, and deep resequencing with at least 50 × depth would be necessary for that purpose^35^. In the future, deep sequencing will allow us to study GC events and their mechanisms in great detail. Combining the single-cell technique in plants with whole-genome sequencing enables the inference of the haplotype of individual pollen grains or ovules based on genotyping of corresponding tetrad cells or polar cells^19^.

## Methods

### Material preparation

F_1_ individuals from the cross of SK and Zheng58 inbred lines were harvested in Hainan in 2012. The F_1_ individuals were planted in Wuhan in the summer of 2013. Immature tassels were harvested before they had emerged, and were maintained in water. A RIL population with 204 families derived from the same two SK and Zheng58 parents was developed and genotyped with the maize SNP50 chip^27^. A high-density linkage map with 13,703 polymorphic markers containing 2,486 unique bins was constructed.

### Isolation and lysis of single cells in tetrads

We used a thin glass pipette system and Programmable Microinjector PM 2,000 (MDI, South Plainfield, USA) to isolate single tetrads and single microspores, and to destroy the cell wall. During the isolation on microscope slide, the tetrad samples were submerged in isolation buffer (27% D-sorbitol solution). Tetrads were extracted from anthers onto a glass slide and single tetrads were separated individually into a new drop of solution. By aspirating a single tetrad in and out repeatedly, the four microspores were separated (Fig. 1a–g). The cells were then aspirated into PCR tubes filled with PBS buffer from the REPLI-g Single Cell Kit (QIAGEN, Hilden, Germany). These tubes were kept on ice to ensure that DNA would not be degraded. All four single microspores from 56 complete tetrads (*n*=26 versus *n*=30 from the two F_1_ individuals, respectively) were isolated from the tassels of two F_1_ individuals with identical genotypes.

### Single-cell DNA whole-genome amplification

We used the QIAGEN REPLI-g Single Cell Kit to lyse single cells and amplify their DNA by MDA^24^ following the standard protocol. The whole-genome amplification products were submitted for detection and whole-genome sequencing.

### Quality control of whole-genome amplification products

To assess the quality and coverage of whole-genome amplification products, we chose 10 polymorphic molecular markers, one from each of the 10 maize chromosomes (Supplementary Table 5). In theory, the genotype of these markers should exhibit Mendelian segregation as 2:2 in the four microspores from the same tetrad. Low-quality product DNA samples with abnormal or undetectable segregation in more than two of the 10 markers were discarded. A total of 180 of 224 single-cell whole-genome amplification samples showed expected segregation patterns for more than eight of the 10 markers and were selected for further analysis (Supplementary Fig. 1). We picked 24 tetrads (*n*=7 versus *n*=17 form the two F_1_ individuals, respectively) for which all four microspores had high-quality whole-genome amplification for whole-genome sequencing. Of these, 12 tetrads were randomly selected and also sequenced using the maize SNP chip with 3,074 SNPs, for comparison purposes (Supplementary Table 1).

### Whole-genome sequencing and SNP calling of haploids

The DNA samples of 96-single cells from 24 tetrads were used to construct Illumina Standard DNA Libraries by TruSeq DNA Sample Prep v2 Kit (Illumina, San Diego, USA) and were subjected to pair-end sequencing on Illumina Hiseq 2,000 platform. We obtained 4.05 billion raw reads from 96 cells. Low-quality bases and reads were removed by Trimmomatic 3.0 (ref. 50). We mapped those reads to the maize reference genome AGPv3 by Burrows-Wheeler Alignment tool^51^ and samtools^52^. SNP calling was conducted by samtools mpileup^52^. The detailed protocol for single-cell isolation and sequencing for maize microspores from tetrads can be downloaded from http://www.maizego.org/Resources.html.

### Parental SNP calling

SK leaf DNA was extracted by Plant Genomic DNA Kit (TIANGEN, Beijing, China) and used for preparing Illumina Standard DNA Library, which was then sequenced on Illumina Hiseq 2,000 platform. The sequencing data of Zheng58 (‘SRR449340’, ‘SRR449342’ and ‘SRR449343’) were downloaded from the NCBI SRA database (http://www.ncbi.nlm.nih.gov/sra/). For the parent SK, up to 8.0 × sequencing coverage of the whole genome was obtained, while the genome sequence data of the parent Zheng58 has 15.7 × depth coverage for SNP calling. Both data sets then were filtered by Trimmomatic 3.0 (ref. 50), mapped to reference genome by SOAP2 (ref. 53 and subjected to SNP calling by SOAPsnp^54^. The following criteria were used for SNP calling: (1) Q scoreowing(2) reads depthg criteria were used for(3) depth uniquely mapped reads for calling SNPling: tection for maand (4) only homozygous SNPs were considered. Parental genotypes were then compared and polymorphic SNPs (dSNPs) identified between them. We required that the minimum distance between two SNPs to be no <4 bp. Ultimately, 1,269,588 raw SNPs were obtained by comparing parental whole-genome sequences (Supplementary Table 2).

### SNP filtering at the population level

Further, filtration of raw SNPs was conducted as follows: (1) minor allele frequency of SNPs <0.1 in the whole population were removed; (2) SNPs detected in <10 microspores were removed; and (3) singletons with different haplotypes, containing no >20 SNPs, identified in <5 microspores and thus with potentially low reliability were removed. In total, 599,154 high-quality SNPs were obtained (Supplementary Table 2) and were directly used to assign true haplotypes of the 96 microspores.

### Crossover analysis at the genome level

We downloaded the annotated maize genome ‘Zea_mays.AGPv3.21.gff3’ from www. maizegdb.org and extracted physical coordinates of AGT and UGA sites for 53,648 qualified transcripts. We searched for the closest transcript for each CO. The location of COs was considered to be the centre of CO tract. For simplicity, we then adjusted the length between ATG and UGA in each gene model to be the average gene length in maize (2,500 bp calculated based on B73 genome). To avoid possible errors, we only focused on 581 (62.9%) of the CO tracts of >100 Kb in length, to draw a frequency plot of distance between CO and the ATG site. As negative controls, 581 random locations per set were simulated five times (Fig. 2c). At a smaller scale, we focused on COs within tracts of <10 Kb (234, 25.3%) to determine the exact location of COs relative to the gene models by the histogram (Fig. 2d).

### COCT validation by Sanger sequencing

Five COCTs were randomly selected from those fine-mapped to a narrow genomic region of <4 Kb, and were validated by Sanger sequencing of PCR products. Primers are provided in Supplementary Table 5.

### Crossover interference based on CoC

CoC was evaluated as the ratio of observed over expected double CO. It was calculated at both the single-microspore level and tetrad level. Crossovers were counted in a 1-Mb sliding window. The expected double CO rate can be obtained by multiplying two single CO rates from any two given sites. The CoC can be calculated as the ratio of observed double CO rate from the two corresponding sites to the expected rate. Finally, the average CoC of sites the same distance apart was calculated (Fig. 4a).

## Author contributions

J.Y. designed and supervised this study. X.L. performed the experiments and analysed the data with the help from L.L. and J.Y. X.L., L.L. and J.Y. prepared the manuscript.

## Additional information

**Accession codes:** The sequencing data for cells isolated from maize tetrads have been deposited in the NCBI Sequence Read Archive under accession code SRP047362.

**How to cite this article:** Li, X. *et al*. Dissecting meiotic recombination based on tetrad analysis by single-microspore sequencing in maize. *Nat. Commun.* 6:6648 doi: 10.1038/ncomms7648 (2015).

## Supplementary Material

Supplementary Information Supplementary Figures 1-3, Supplementary Tables 1-5 and Supplementary References

Supplementary Dataset 1 Single microspore sequencing data summary

Supplementary Dataset 2 All COs at the tetrad level

Supplementary Dataset 3 CO count at the single cell level

## Figures and Tables

**Table 1:** Comparison of recombination patterns among species involved in reproductive cell sequencing.

**Species**	**Genotyping method**	**CO number per individual (or cell)**	**CO distribution**	**CO interference**	**Chromatid interference**
*Human*	Single reproductive cell sequencing^17^,^18^,^19^	22.8 (male)^17^, 26 (male)^18^, 43 (female)^19^	Preference to stay away from transcripts^18^,^19^,^30^	Positive <20 Mb (female)^19^/45 Mb (Male)^18^	Weak and negative^19^
*Yeast*	Sequencing clones of meiotic progenies from the same ascus^10^,^11^	45 (ref. 10)	Concentration to gene promoters^28^	Positive <30 cM^39^	Conflict of different reports^42^,^43^,^44^,^45^
*Arabidopsis*	Sequencing four progenies crossed by one *qrt1* mutant tetrad^35^,^46^	5.0 (male)^35^	Concentration to gene promoters^29^	Positive <50 cM^41^	No data
*Maize*	Sequencing single microspores from the same tetrad^*^	19.3 (male)^*^	Concentration to 5′ end of transcripts^*^	Positive <10 Mb^*^, and strong negative above 10 Mb^*^, varied among maize populations^6^	Weak and complex interference^*^
In present study.					
