# Supplementary material for: Dissecting meiotic recombination based on tetrad analysis by single-microspore sequencing in maize
Source: Nat Commun. 2015 Mar 24;6:6648. doi: 10.1038/ncomms7648 (PMC4383000; doi:10.1038/ncomms7648)
Supplement: Supplementary Information — Supplementary Figures 1-3, Supplementary Tables 1-5 and Supplementary References [file ncomms7648-s1.pdf]

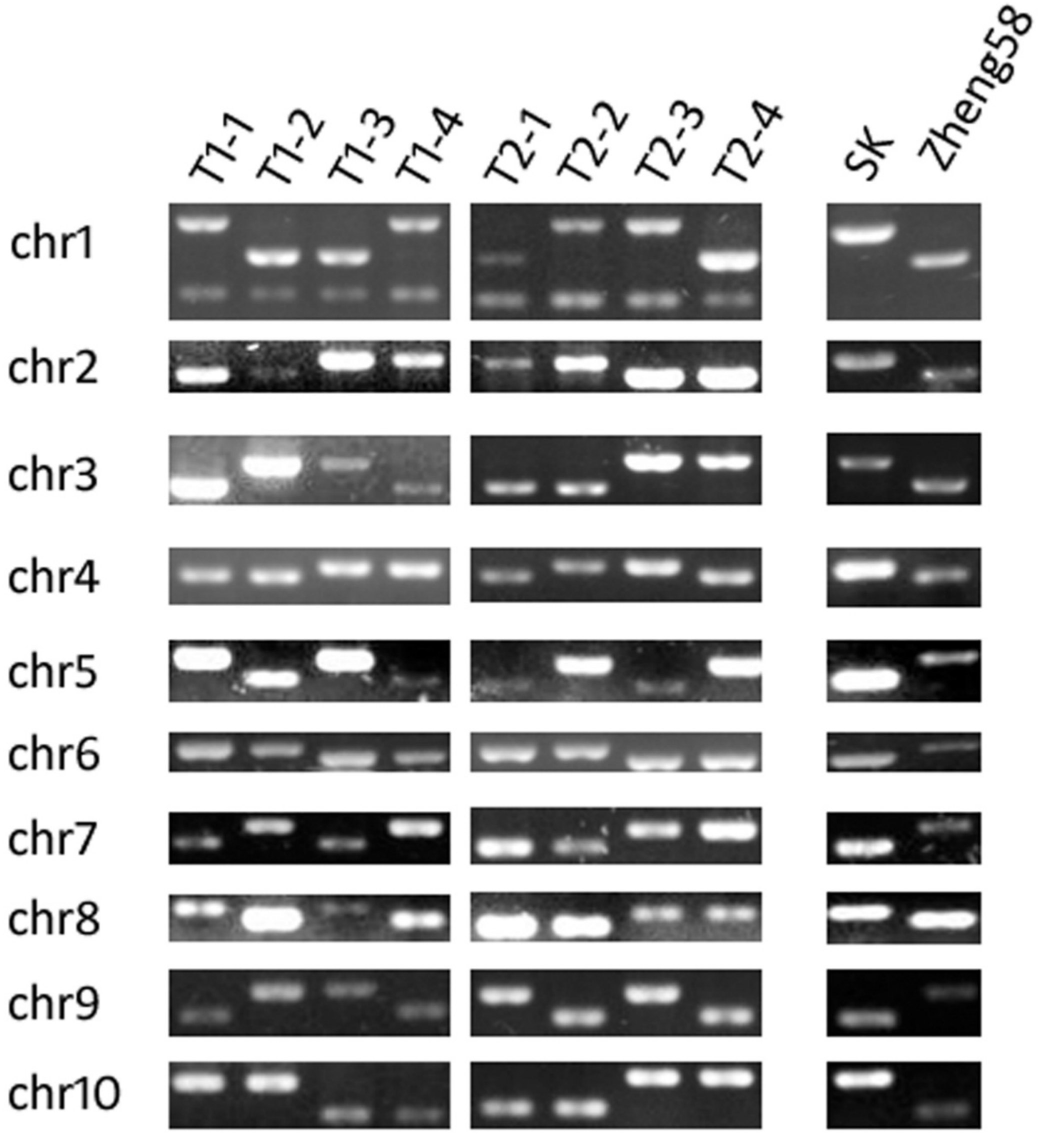

Supplementary Figure 1 | Examples of detection of MDA products based on molecular markers. To assess quality of whole-genome amplification by MDA, we selected 10 markers (one per chromosome), segregating between the two parents (SK and Zheng58). High-quality of MDA would be indicated by the proper segregation ratio (2:2) of tested markers. T1-1, T1-2, T1-3 and T1-4 are the four microspores of tetrad 1, respectively. T2-1, T2-2, T2-3 and T2-4 are the four microspores of tetrad 2, respectively.

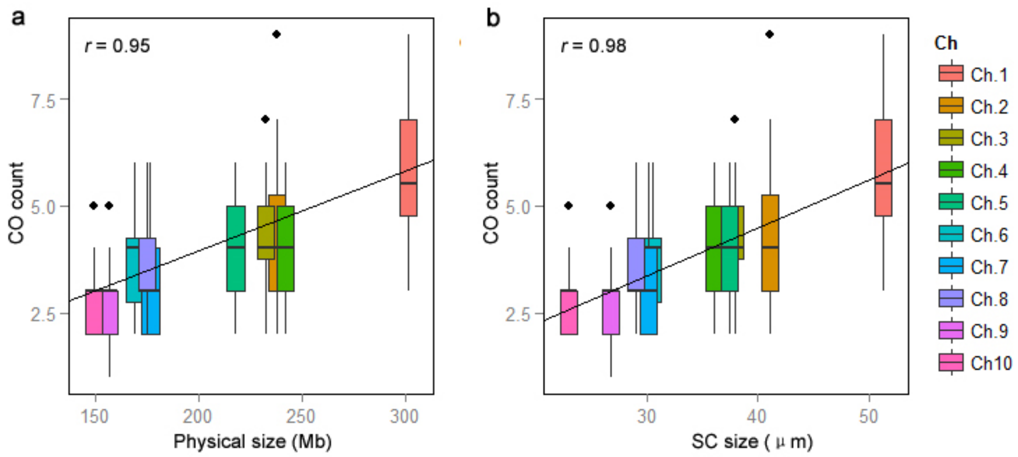

Supplementary Figure 2 | Relationships between CO counts in each chromosome, chromosome length and synaptonemal complex (SC) size. The positive correlations between CO count and physical size (a) and SC size (b) are presented. *r* is the Pearson correlation coefficient. The error bars mark the range from 5th and 95th percentile of the total sets (a, b). SC size was adopted from maize KYS inbred[1].

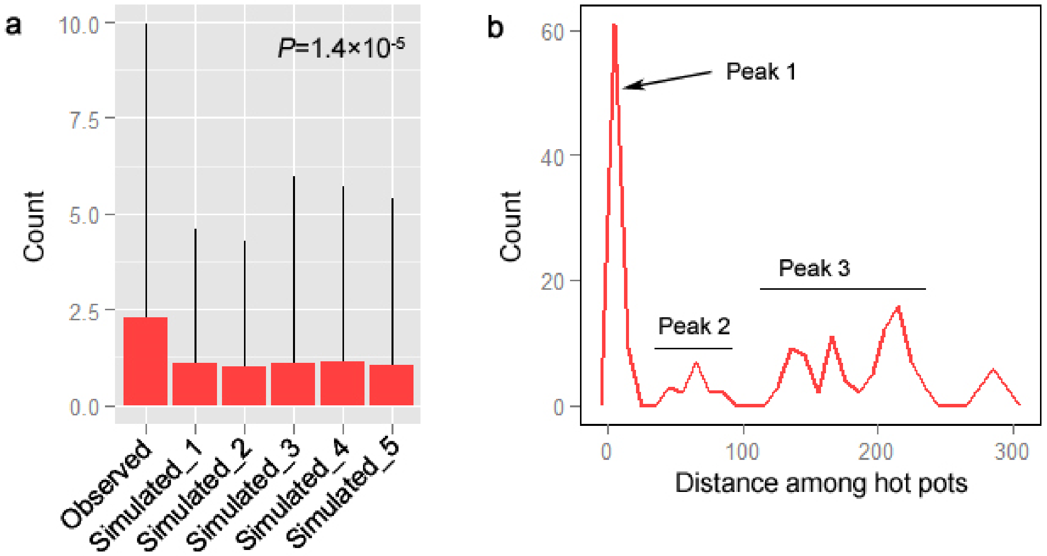

Supplementary Figure 3 | Crossover hot spots. Totally, 64 crossover hot spots were characterized, with at least 5 COs per 3 Mb. (a) The CO count in regions 10 Mb away from these hot spots is significantly more than those simulated, which is shown as the Peak 1 in b. Error bars mark the range from 5th and 95th percentile of the total data (a). (b) The distribution of interval distances between pairs of crossover hot spots. Three peaks which may correspond to the peaks in Figure 4B, were observed.

Supplementary Table 1. Validation of 12 tetrads by SNP array

Sample	Non-polymorphic SNPs	Rate	Polymorphic SNPs	Rate	Heterozygous SNPs	Rate
T1-1	1167	59.9%	684	35.1%	14	0.7%
T1-2	1170	60.1%	651	33.4%	35	1.8%
T1-3	1140	58.5%	722	37.1%	1	0.1%
T1-4	1046	53.7%	695	35.7%	0	0.0%
T2-1	1172	60.2%	645	33.1%	52	2.7%
T2-2	1074	55.1%	740	38.0%	1	0.1%
T2-3	1122	57.6%	749	38.4%	1	0.1%
T2-4	1123	57.6%	740	38.0%	0	0.0%
T3-1	1103	56.6%	729	37.4%	0	0.0%
T3-2	1090	56.0%	732	37.6%	1	0.1%
T3-3	1041	53.4%	632	32.4%	39	2.0%
T3-4	1172	60.2%	614	31.5%	67	3.4%
T4-1	1169	60.0%	618	31.7%	64	3.3%
T4-2	1108	56.9%	735	37.7%	0	0.0%
T4-3	1087	55.8%	720	37.0%	0	0.0%
T4-4	1038	53.3%	681	35.0%	2	0.1%
T7-1	1095	56.2%	720	37.0%	3	0.2%
T7-2	1083	55.6%	705	36.2%	1	0.1%
T7-3	1101	56.5%	720	37.0%	1	0.1%
T7-4	1139	58.5%	662	34.0%	21	1.1%
T9-1	781	40.1%	519	26.6%	0	0.0%
T9-2	1128	57.9%	743	38.1%	0	0.0%
T9-3	1129	58.0%	747	38.3%	1	0.1%
T9-4	1124	57.7%	737	37.8%	4	0.2%
T10-1	966	49.6%	589	30.2%	21	1.1%
T10-2	1080	55.4%	727	37.3%	0	0.0%
T10-3	1106	56.8%	714	36.7%	2	0.1%
T10-4	1079	55.4%	729	37.4%	1	0.1%
T11-1	993	51.0%	654	33.6%	0	0.0%
T11-2	1015	52.1%	678	34.8%	0	0.0%
T11-3	989	50.8%	663	34.0%	0	0.0%
T11-4	1074	55.1%	721	37.0%	0	0.0%
T12-1	1062	54.5%	688	35.3%	0	0.0%
T12-2	1072	55.0%	713	36.6%	1	0.1%
T12-3	1121	57.5%	751	38.6%	0	0.0%
T12-4	1122	57.6%	746	38.3%	0	0.0%
T15-1	1137	58.4%	742	38.1%	0	0.0%
T15-2	1122	57.6%	747	38.3%	0	0.0%
T15-3	1089	55.9%	732	37.6%	0	0.0%
T15-4	1106	56.8%	735	37.7%	1	0.1%
T17-1	1143	58.7%	759	39.0%	0	0.0%

T17-2	1141	58.6%	761	39.1%	0	0.0%
T17-3	1134	58.2%	747	38.3%	0	0.0%
T17-4	1113	57.1%	747	38.3%	0	0.0%
T22-1	1108	56.9%	742	38.1%	1	0.1%
T22-2	1124	57.7%	734	37.7%	0	0.0%
T22-3	1116	57.3%	742	38.1%	0	0.0%
T22-4	1165	59.8%	691	35.5%	30	1.5%
SK-1	1165	59.8%	775	39.8%	0	0.0%
SK-2	1166	59.9%	775	39.8%	0	0.0%
Zheng58-1	1173	60.2%	775	39.8%	0	0.0%
Zheng58-2	1142	58.6%	766	39.3%	1	0.1%

Supplementary Table 2. SNPs count in 10 chromosomes

	chr1	chr2	chr3	chr4	chr5	chr6	chr7	chr8	chr9	chr10	total
Raw SNPs	176983	137074	154878	156364	129244	99226	115994	115918	95289	88618	1269588
High-quality SNPs	82710	66738	75737	72921	56536	48245	53098	54330	46141	42698	599154

Supplementary Table 3. CO counts at the tetrad level in 10 chromosomes

Tetrad	Individual (Donor)	Chr1	Chr2	Chr3	Chr4	Chr5	Chr6	Chr7	Chr8	Chr9	Chr10	All chr.
3	1	5	4	4	2	3	3	3	3	2	2	31
4	1	3	3	4	3	2	2	2	5	3	2	29
5	1	3	3	2	3	3	2	2	2	2	2	24
6	1	5	4	4	4	4	4	2	3	1	3	34
7	1	4	3	3	4	2	3	2	2	3	3	29
8	1	6	5	5	5	3	5	3	4	3	3	42
9	1	3	4	3	3	5	2	4	3	3	2	32
1	2	7	2	4	4	5	5	5	5	3	3	43
2	2	5	7	2	6	4	4	3	3	4	3	41
10	2	6	4	6	3	3	2	3	3	2	2	34
11	2	8	5	6	5	6	6	2	4	3	3	48
12	2	9	4	6	5	5	5	2	5	5	3	49
13	2	7	3	5	4	5	5	3	4	3	5	44
14	2	5	4	5	3	4	3	2	3	2	2	33
15	2	6	5	3	2	5	2	3	3	2	3	34
16	2	5	6	4	5	5	6	4	2	2	3	42
17	2	7	9	7	5	4	4	3	6	3	2	50
18	2	6	4	4	5	4	4	5	5	3	3	43
19	2	5	6	2	5	5	4	6	2	2	2	39
20	2	8	6	4	3	4	4	4	5	2	4	44
21	2	6	3	5	5	5	4	5	2	3	3	41

22	2	4	4	4	4	3	4	4	4	5	3	39
23	2	4	3	5	3	6	3	4	4	3	2	37
24	2	7	6	5	4	6	2	4	3	3	2	42

Supplementary Table 4. Comparison of CO counts between two different F_1 individuals

	Tetrad	Chr1	Chr2	Chr3	Chr4	Chr5	Chr6	Chr7	Chr8	Chr9	Chr10	All
all individuals	CO_total	134	107	102	95	101	88	80	85	67	65	924
all individuals	CO_average (tetrad level)	5.6	4.5	4.3	4.0	4.2	3.7	3.3	3.5	2.8	2.7	38.5
all individuals	CO_average (cell level)	2.8	2.2	2.1	2.0	2.1	1.8	1.7	1.8	1.4	1.4	19.3
Individual 1	CO_total	29	26	25	24	22	21	18	22	17	17	221
Individual 1	CO_average (tetrad level)	4.1	3.7	3.6	3.4	3.1	3.0	2.6	3.1	2.4	2.4	31.6
Individual 1	CO_average (cell level)	2.1	1.9	1.8	1.7	1.6	1.5	1.3	1.6	1.2	1.2	15.8
Individual 2	CO_total	105	81	77	71	79	67	62	63	50	48	703
Individual 2	CO_average (tetrad level)	6.2	4.8	4.5	4.2	4.6	3.9	3.6	3.7	2.9	2.8	41.4
Individual 2	CO_average (cell level)	3.1	2.4	2.3	2.1	2.3	2.0	1.8	1.9	1.5	1.4	20.7
RIL	CO_total	1419	1028	929	917	868	679	733	689	636	418	8316
RIL	CO_average	7.0	5.0	4.6	4.5	4.3	3.3	3.6	3.4	3.1	2.0	40.8

Supplementary Table 5. PCR primers used in this study

Name	Sequence	Note
CO-1(section 1)	5'-CTATGCCGGTAAGGTTGGATTC-3'/5'-CCGAGTTGTTCATACGAGACT-3'	Figure 3a
CO-1(section 2)	5'-GCACCCTTATGAGTTTCTACC-3'/5'-GGCTCTGCTGCCTGACTTAT-3'	Figure 3a
CO-2	5'-GGCGTGCTAGGACAGCAAAC-3'/5'-GTCTACACCACTCACAATGGC-3'	Figure 3b
CO-3	5'-CACAAACCGGGACTTGCTAC-3'/5'-AAAGAGCGCCAAGTACCAGC-3'	Figure 3c
CO-4	5'-CAGTGACTTGTACGGCTCCC-3'/5'-CACAAATCACGCCTCAGAGC-3'	Figure 3d
CO-5	5'-TCCGTGACCACCTTAGCAAC-3'/5'-TCTCGGTTGGATTCGCCTTC-3'	Figure 3e
maker_chr1	5'-AAGTGGTGAGGTAAGCCTGC-3'/5'-ATAGGAGACACCCTGGGCAT-3'	Supplementary Figure 1
maker_chr2	5'-CTCTTCCAATCGGGTTTGC-3'/5'-AATTGCACATAACAGAGGCG-3'	Supplementary Figure 1
maker_chr3	5'-CTCAGGAGGAGGAAATGTGG-3'/5'-CTTCTGTCCGTGAAGGATGG-3'	Supplementary Figure 1
maker_chr4	5'-AAGAACAGCATTGTCGTCACC-3'/5'-GTCCAGCGTCAGAGCTTACC-3'	Supplementary Figure 1
maker_chr5	5'-AAAGCACTTACATCATGGGAAAC-3'/5'-TTGGTGTAGCTCCGATTTG-3'	Supplementary Figure 1
maker_chr6	5'-CATAGTCCGATCTTGGTGACG-3'/5'-CATACAGGGAGTCACGGTCC-3'	Supplementary Figure 1
maker_chr7	5'-AGCACCAGGAAGTTGTGAGG-3'/5'-CCAACTCGATACGAAGAGCC-3'	Supplementary Figure 1
maker_chr8	5'-TCCAAGTCCCATGGCAGAAC-3'/5'-CCATGCTAGCTGATCGTCGT-3'	Supplementary Figure 1
maker_chr9	5'-CCCGAGTTGCATGGAAGACA-3'/5'-CGTGCGTTTGAATTGGCTGA-3'	Supplementary Figure 1
maker_chr10	5'-CCTTCTAATTAAAGTCAAAGCCA-3'/5'-CAACACCCAACATCCGTGCT-3'	Supplementary Figure 1

Supplementary References

1. Falque, M., Anderson, L. K., Stack, S. M., Gauthier, F. & Martin, O. C. Two types of meiotic crossovers coexist in maize, *Plant Cell* **21**, 3915-3925 (2009).
